# Case report: Recurrence of anti-myeloperoxidase pauci-immune crescentic glomerulonephritis in a kidney transplant recipient; potential association with HLA antigens and seropositivity?

**DOI:** 10.3389/fimmu.2026.1713188

**Published:** 2026-06-17

**Authors:** Woojin James Chon, Choli Hartono, Elena-Rodica Vasilescu, David Serur, Ibrahim Batal

**Affiliations:** 1 Department of Medicine, Hackensack Meridian School of Medicine, Hackensack, NJ, United States; 2 Department of Organ Transplantation, Hackensack University Medical Center, Hackensack, NJ, United States; 3 Department of Pathology and Cell Biology, Columbia University Irving Medical Center, New York, NY, United States

**Keywords:** ANCA - associated vasculitis, kidney, pathology, recurrent glomerular disease, transplantation

## Abstract

Anti-myeloperoxidase (anti-MPO) antineutrophil cytoplasmic antibodies-associated vasculitis (AAV) manifests histologically as pauci-immune crescentic glomerulonephritis. Recurrent anti-MPO AAV after transplantation is rare and poorly understood. In this report, we describe a case of early recurrence of anti-MPO AAV in the kidney allograft of a patient who shared potentially permissive HLA antigens with the donor and who had detectable anti-MPO antibodies post-transplantation. We also share our experience with 13 additional cases of anti-MPO AAV in the native kidney who underwent kidney transplantation but did not develop recurrent disease. Clinicopathologic correlates, focusing on the potential association with post-transplant anti-MPO levels and permissive HLA antigens in the donors and recipients, are discussed. This report provides a brief examination of the immunopathologic features of anti-MPO AAV in the kidney allograft and underscores the potential importance of close monitoring for disease recurrence in patients presumed to be at increased risk for such complication.

## Introduction

Antineutrophil cytoplasmic antibodies (ANCA)-associated vasculitis (AAV) is a rapidly progressive pauci-immune crescentic glomerulonephritis (GN) that can frequently lead to native kidney failure (1, 2). Typically, AAV has antibodies against myeloperoxidase (MPO) and/or proteinase 3 (PR3) (2). HLA-DR4 and HLA-DQ7 antigens, which are in linkage disequilibrium, have been implicated in both anti-MPO AAV and anti-PR3 AAV (3), HLA-DQ9 and HLA-DR9, that are in linkage disequilibrium, are associated with anti-MPO AAV (4, 5), and HLA-DPB1*04:01 has been linked to anti-PR3 AAV (6). Patients with anti-MPO AAV have lower risk of relapse and more favorable survival (1). Post-transplant recurrence of AAV in the kidney allograft is rare, especially in the modern era of immunosuppressive regimens (7). We present a case of early recurrence of AAV in a kidney transplant recipient who shared HLA-DR4 and HLA-DQ7 antigens with her deceased donor, was on belatacept-based immunosuppression maintenance, and had elevated anti-MPO antibodies at the time of recurrence.

## Case description

Sixty-three-year-old Hispanic female, with past medical history of hypertension, pre-diabetes, and hypothyroidism, developed kidney injury secondary to anti-MPO AAV [MPO > 100 antibody index (AI); negative value <1 AI]. Despite treatment with rituximab and steroids, the patient developed native kidney failure two years later. One year later, at the age of 66 years, the patient underwent a kidney transplantation from a three-antigen mismatched (A, B, DR: 0-6) deceased donor (54 year-old male), who expired from cardiovascular accident and donated the kidney after cardiac death. Donor’s terminal serum creatinine (sCr) was 0.6 mg/dL and Kidney Donor Profile Index was 49%.

Donor HLA typing: A2, A2, B7, B51, DR4, DR13, DQ6, DQ7

Recipient HLA typing: A2, A24, B39, B52, DR4, DR14, DQ7, DQ8

Cold ischemia time was 24 hours, and warm ischemia time was 26 minutes. The virtual crossmatch was negative and no pre-transplant circulating anti-HLA donor-specific antibodies (DSA) were detected. Induction therapy consisted of thymoglobulin (total 4.5 mg/kg) and maintenance immunosuppressive regimen comprised belatacept, mycophenolate sodium, and prednisone.

Post-transplant course was complicated by slow graft function and low-level BK viremia (45 IU/mL 4-weeks post-transplantation). Five weeks after transplantation, the patient presented for an allograft biopsy in the setting of persistently elevated sCr of 3.1 mg/dL. Physical examination showed blood pressure: 150/73 and body mass index of 23 without lower extremity edema. Laboratory workup revealed proteinuria: 30 mg/dL/1+ albumin, mildly elevated cell free DNA (1.3%), red blood cell count: 2.6 x 10^6^/µL (reference range: 4.0-5.1 x 10^6^/µL), white blood cell count: 7.0 x 10^3^/µL (reference range: 4.0-11.0 x 10^3^/µL), platelet count: 270 x 10^3^/µL (reference range: 135–430 x 10^3^/µL), urine culture growing Enterobacter cloacae complex, and urine sediment with 21–50 RBCs/hpf and 0–5 WBCs/hpf. Allograft ultrasound was unremarkable.

### Kidney allograft biopsy

**Figure 1:**
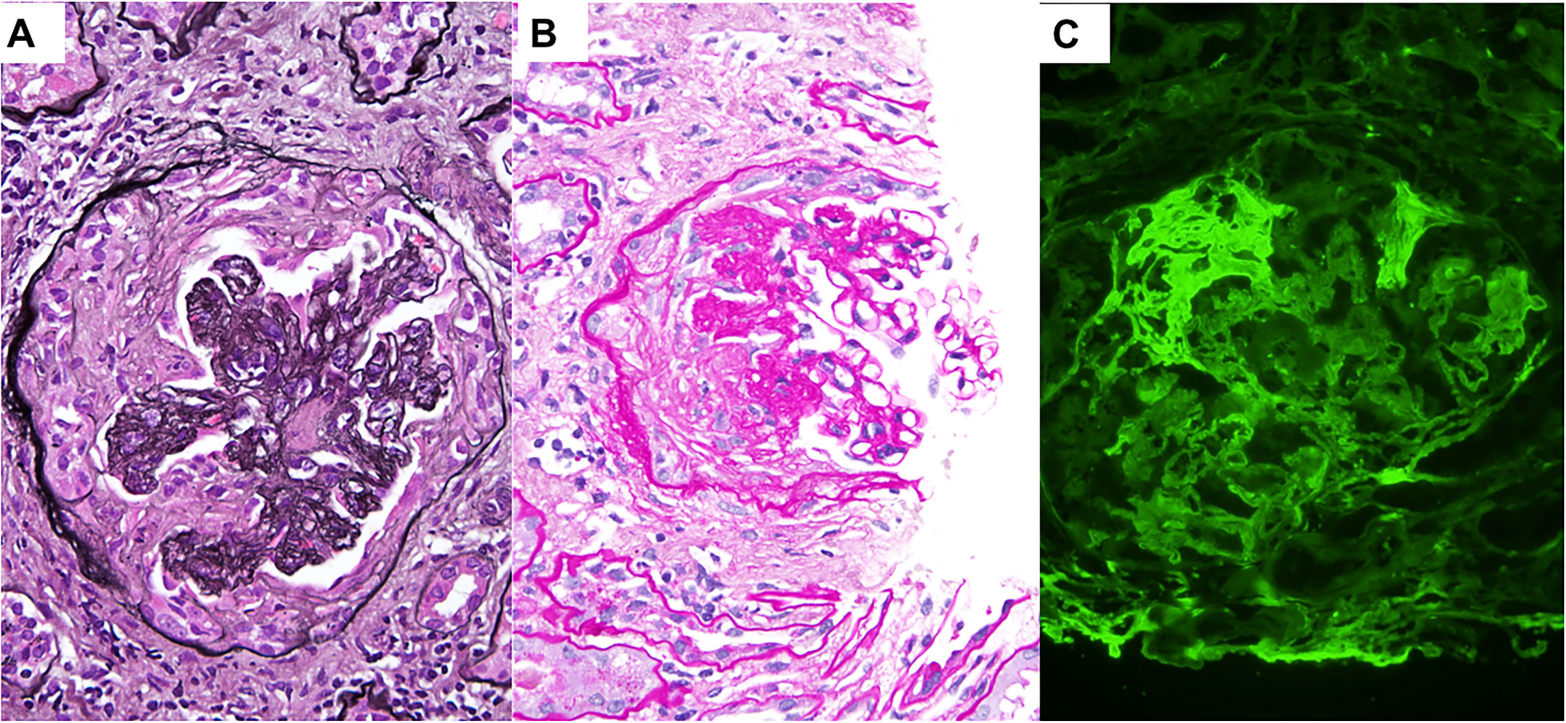
Histologic findings in the kidney allograft biopsy. **(A)** A glomerulus with a cellular crescent (Jones methenamine silver, original magnification x400). **(B)** A glomerulus with a fibrous crescent (Periodic Acid-Schiff, original magnification x400). **(C)** Positive fibrin staining in Bowman’s space, indicative of an active crescent (immunofluorescence, original magnification x400).

Sampling for light microscopy contained 21 glomeruli, of which 8 were globally sclerotic. Of the 13 non-globally sclerotic glomeruli, 1 displayed a circumferential cellular crescent (**Figure 1A**), 3 demonstrated fibrocellular crescents, and 3 showed fibrous crescents (**Figure 1B**). Unaffected glomeruli revealed focal mild mesangial sclerosis without significant mesangial hypercellularity, endocapillary hypercellularity, or glomerular basement membrane duplication. There was moderate tubulointerstitial scarring, mild interstitial inflammation with rare foci of moderate tubulitis, focal tubular generative changes manifested as simplification of the lining epithelium and luminal ectasia, scattered tubules containing red blood cell casts, focal severe arteriosclerosis, and moderate arteriolosclerosis without significant peritubular capillaritis. Immunoperoxidase staining for SV40 was negative. The immunofluorescence showed fibrin staining in Bowman’s space of one of four glomeruli (**Figure 1C**). Beside fibrin staining, immunofluorescence revealed negative C4d staining in peritubular capillaries and no significant glomerular staining for other immune reactants.

In summary, the kidney allograft biopsy findings were consistent with recurrent pauci-immune focal crescentic GN. The focal tubulointerstitial inflammation and tubular degenerative changes were mainly attributed to the aggressive GN and the red blood cell casts, respectively.

### Follow-up

Laboratory work-up revealed elevated anti-MPO antibodies (>800 AI), with negative anti-PR3 and anti-glomerular basement membrane antibodies. The patient was treated with pulse steroids (500mg x 3 doses, Prednisone 10 mg po daily), and Rituximab (375mg per m_2_, x 4 doses). One month later, sCr decreased to 2.7 mg/dL, urine protein-to-creatinine ratio was 0.7 g/g, and urine sediment showed >50 RBCs/hpf and 6–10 WBCs/hpf. Five months later, urine microscopy showed decreased microhematuria to 3–5 RBCs/hpf, dipstick revealed 100 mg/dL of albumin, and BKV DNA was undetectable in the plasma. Five months later, anti-MPO antibodies remained detectable although decreased to 237 AI. At last follow-up (approximately one-year post-biopsy), sCr decreased further to 1.6 mg/dL.

### Additional investigations

This case of recurrent anti-MPO AAV, which was associated with HLA-DR4 and HLA-DQ7 antigens in both donor and recipient and with positive anti-MPO antibodies post-transplantation, turned our attention to investigate the association of HLA antigens and circulating anti-MPO antibodies with recurrent diseases. We retrospectively reviewed our pathology database for kidney transplant recipients who developed native kidney failure secondary to anti-MPO AAV, received kidney transplantation from 2005 to 2025, and had kidney allograft or native biopsies reviewed at Columbia University Irving Medical Center. This study was approved by the Institutional Review Board.

Thirteen additional patients were identified, none of which developed recurrent disease (**Table 1**). Five of the recipients (38%) had HLA-DQ7 antigens and four of these (31%) shared DQ7 with the donor, but none of them showed positive anti-MPO antibodies after transplantation (3 had negative tests and 1 was never assessed for anti-MPO antibodies). Three recipients (23%) had HLA-DR4 antigens, of which one (8%) shared this antigen with the donor, but this patient had negative anti-MPO antibodies post-transplantation. Two recipients (15%) had HLA-DQ9 antigens, of which one (8%) shared this antigen with the donor, but this patient also had negative anti-MPO antibodies post-transplantation. None of the recipients had HLA-DR9 antigen.

**Table 1:** Cases of recipients of kidney allograft after native kidney failure attributed to anti-MPO ANCA associated vasculitis.

Patients#	Recipient HLA Ags of interest	Donor HLA Ags of interest	Post-transplant anti-MPO testing	Type of transplant	Immunosuppression (induction; maintenance)	Recurrence
1	None	None	Negative	Living	Thymoglobulin; tacrolimus, mycophenolic acid	No
2	None	DR4	NA	Deceased	Thymoglobulin; tacrolimus, mycophenolic acid	No
3	DQ7	DQ7	Negative	Living)	Basiliximab; tacrolimus, mycophenolic acid	No
4	None	None	Positive(up to 91 AU/mL)	Living	Thymoglobulin; tacrolimus, mycophenolic acid	No
5	None	None	Positive(up to 38 AU/mL)	Deceased	Thymoglobulin; rapamycin, mycophenolate mofetil, prednisone	No
6	DQ9	DQ9	Negative	Deceased	Thymoglobulin; tacrolimus, mycophenolic acid	No
7	DQ7	DQ7	Negative	Living	Thymoglobulin; tacrolimus, mycophenolic acid	No
8	DR4	DQ7	NA	Deceased	Thymoglobulin; tacrolimus, mycophenolate mofetil, prednisone (converted to belatacept-based regimen two months post-transplantation)	No
9	DR4/DQ7	DR4/DQ7	Negative	Living	Thymoglobulin; tacrolimus, mycophenolic acid	No
10	None	None	Negative	Deceased	Thymoglobulin; tacrolimus, mycophenolic acid (converted to belatacept-based regimen five days post-transplantation)	No
11	DR4/DQ7	DQ7	NA	Living)	Thymoglobulin; tacrolimus, mycophenolate mofetil	No
12	None	DR4	NA	Deceased	Thymoglobulin; tacrolimus, mycophenolic acid	No
13	DQ7, DQ9	None	NA	Deceased	Thymoglobulin; belatacept, mycophenolic acid, prednisone	No
Current case	**DR4/DQ7**	**DR4/DQ7**	**Positive** **(>800 AI/mL)**	Deceased	**Thymoglobulin; belatacept, mycophenolate sodium, prednisone**	**Yes**

Ags, antigens.Bold value is used to label the index case, which was the only case developing post-transplant recurrence.

Regarding post-transplant anti-MPO levels, two patients had positive post-transplant antibodies, but none of these two recipients nor their donors had any of the potentially permissive HLA antigens (DQ7, DQ9, DR4 or DR9) (**Table 1**).

## Discussion

We present a challenging case of a patient with a history of AAV who developed early recurrence post-transplantation after receiving a kidney allograft from a deceased donor and while was maintained on belatacept-based immunosuppression regimen.

In general, cumulative evidence supports that immune and genomic factors are important contributors to the development of glomerular diseases in the native kidneys and to their recurrence post-transplantation (8). Furthermore, the role of permissive donor HLA antigens in recurrent diseases after transplantation has been suggested in a subset of glomerular diseases, including membranous nephropathy (9) and diffuse podocytopathy (10).

In the native kidney, genome-wide association studies have shown that anti-MPO AAV is associated with certain HLA antigens in the DR and DQ regions, such as DR4, DR9, DQ7, and DQ9 (3–5). Another study has shown that reappearance of anti-MPO autoantibodies is a risk factor for relapsing disease (11).

In the transplant settings, recurrence of AAV is rare (7) when compared to other glomerular diseases such as C3 glomerulopathy (12) and even to IgA nephropathy (13) or membranous nephropathy (14). However, the poorly understood risk factors for recurrent AAV, together with the lack of reliable biomarkers, make it difficult to diagnose and treat disease recurrence in a timely manner.

To further our understanding of the immune and hereditary risk factors of post-transplant recurrence, we identified 14 patients who developed native kidney failure secondary to anti-MPO AAV and received kidney transplantation and we focused our assessment on HLA antigens of interest (DR4, DR9, DQ7, DQ9) as well as post-transplant assessment of anti-MPO antibodies (**Table 1**). Only one (7%) of these 14 patients developed recurrent disease. Notably, this was the only patient who fulfilled the following (A) shared HLA antigens frequently associated with AAV with the donor (in this case DR4 and DQ7) and (B) had detectable anti-MPO antibodies post-transplantation. Six (43%) patients shared HLA antigens of interest with their donors without development of anti-MPO antibodies post-transplantation while two (14%) patients had detectable anti-MPO antibodies after transplantation but did not share potentially permissive HLA antigens with their donors. Remarkably, none of these 8 patients developed recurrent disease. This may suggest that close monitoring of patients with apparently permissible HLA antigens (DR4, DR9, DQ7, DQ9) who shared some of these antigens with their donors for pre-transplant anti-MPO antibodies and for the development of anti-MPO antibodies post-transplantation may be informative in identifying patients at increased risk for recurrence.

Other variables that may potentially influence recurrence include the source of the allograft and immunosuppressive therapy. It is known that ischemia-reperfusion injury, which is expected to be pronounced in deceased donor who donated after cardiac death and had prolonged cold ischemia time, may increase formation of neutrophil extracellular traps (NETs), which can further enhance neutrophil activation, endothelial damage, or both, which may facilitate the development of AAV (15, 16). Furthermore, belatacept-based regimen is often considered less potent than calcineurin inhibitors-based immunosuppressive regimen. In fact, a recently published case described early post-transplant recurrence of AAV in a patient who was on belatacept-based maintenance (17). However, in contrast to our case, the aforementioned case was associated with AAV with dual ANCA positivity (17), which may raise the possibility of drug-induced process. The likelihood of belatacept having a role in the recurrent AAV in our case cannot be ruled-out. However, it is notable that one of 13 non-recurrent cases was similarly maintained with belatacept since transplantation and two additional patients were converted from tacrolimus-based to belatacept-based regimens within the first two months post-transplantation.

Post-transplant treatment of recurrent AAV frequently includes steroids, rituximab, and cyclosporine (7, 17). Our patient was treated with rituximab and steroids and showed slow recovery of allograft function and decreased microhematuria.

In conclusion, we reported a rare case of early recurrent anti-MPO AAV in the kidney allograft that may be affected by genetic similarity (such as sharing specific HLA antigens) between the donor and recipient. Future multicenter larger studies that focus on the importance of immune and genomic factors in recurrent AAV are needed to confirm this preliminary observation after adjusting for other confounding factors, including immunosuppression regimens and source of the allograft. Such studies are crucial to correctly identify the small proportion of patients at risk for AAV recurrence.

## Data Availability

The datasets presented in this article are not readily available but can be made available after obtaining approval by IRB. Requests to access the datasets should be directed to IRB Columbia University through IB.
